# Microscopic and Molecular Identification of *Eimeria* Species in Domestic Rabbits (*Oryctolagus cuniculus*) in Romania

**DOI:** 10.3390/ani15081109

**Published:** 2025-04-11

**Authors:** Beatrice Ana-Maria Jitea (Sîrbu), Sorin Morariu, Mirela Imre, Tiana Florea, Cătălin Bogdan Sîrbu, Iasmina Luca, Simona Dumitru, Gheorghe Dărăbuș

**Affiliations:** 1 Faculty of Veterinary Medicine, University of Life Sciences “King Michael I” from Timisoara, Calea Aradului 119, 300645 Timisoara, Romania; sorinmorariu@usvt.ro (S.M.); mirela.imre@usvt.ro (M.I.); tijana.florea@usvt.ro (T.F.); iasmina.luca@usvt.ro (I.L.); gheorghedarabus@usvt.ro (G.D.); 2 Faculty of Bioengineering of Animal Resources, University of Life Sciences “King Michael I” from Timisoara, Calea Aradului 119, 300645 Timisoara, Romania; 3 Veterinary and Food Safety Department, 4 Surorile Martir Caceu, 300585 Timisoara, Romania; simonagiubega@gmail.com

**Keywords:** coccidiosis, *Eimeria* spp., rabbit, PCR, ITS-1, Romania

## Abstract

Coccidiosis caused by *Eimeria* spp. is a parasitic disease with high morbidity that affects various animal species, including rabbits. In rabbits, eleven *Eimeria* species have been primarily identified through microscopic examination of oocysts; however, this technique has certain limitations that make it challenging to differentiate between *Eimeria* species. The main objective of this study was to morphologically identify *Eimeria* species infecting rabbits in Romania, as well as to confirm these identifications using molecular biology techniques, specifically through the ITS-1 rRNA gene. A total of 236 samples were collected from different regions of Romania, of which 77.56% (183/236) tested positive for at least one *Eimeria* species. The samples were collected from rabbits raised in individual household settings, under various management systems—both in individual cages and in common ground-based enclosures. The areas of origin were chosen randomly, without applying restrictive criteria, specifically to ensure broad representativeness of the farming conditions encountered in practice, regardless of the production system or geographic specificity. The microscopic examination of morphological characteristics identified ten *Eimeria* species, whereas PCR results confirmed the presence of four species. It was not always possible to identify all the species present in a given sample, likely due to the predominant parasitic load and the DNA quantity of each species. The results of this study confirm that ITS-1 is a useful molecular marker for identifying *Eimeria* species in domestic rabbits.

## Simple Summary

Coccidiosis is a common parasitic disease in rabbits, caused by *Eimeria* spp. This study analyzed 236 fecal samples from rabbits in Romania, revealing that 77.56% were infested. Microscopic examination identified ten *Eimeria* species, whereas molecular biology techniques detected only four species. Some species were not detected by PCR due to low DNA quantity. This study demonstrates that the ITS-1 molecular marker is useful for the accurate identification of these parasites in rabbits.

## 1. Introduction

Parasites of the *Eimeria* genus are classified within the subfamily *Eimeriinae* of the phylum *Apicomplexa* [1]. Coccidiosis is a parasitic disease of significant economic importance due to its high morbidity in numerous domestic and wild animal species, including cattle, sheep, goats, pigs, horses, poultry, rabbits, and rodents. Rabbits are susceptible to various parasites, including both ectoparasites and endoparasites. Coccidiosis is considered one of the most significant parasitic diseases affecting domestic rabbits (*Oryctolagus cuniculus*) [2,3]. Coccidiosis in domestic rabbits is caused by eleven *Eimeria* species: *Eimeria intestinalis*, *Eimeria flavescens*, *Eimeria magna*, *Eimeria media*, *Eimeria piriformis*, *Eimeria irresidua*, *Eimeria exigua*, *Eimeria coecicola*, *Eimeria perforans*, *Eimeria vejdovskyi*, and *Eimeria stiedae* [4,5,6,7,8]. High mortality and morbidity have been reported globally, causing significant annual economic losses in the domestic rabbit farming industry, with many farms shutting down due to the severity of the infection [5,9,10]. Various rates have been documented in multiple studies, such as a mortality rate of 85% reported by Omadevuaye et al. [11] and morbidity and mortality rates of 9% and 5%, respectively, as reported by Meek (1943) [12], as well as 48% (Cheeke, 1987) [13] and 64% (Pakes et al., 1994) [14]. In experimental infections, mortality rates of 40% and 80% in young rabbits have been reported by Percy and Barthold (2007) [15].

Typically, *Eimeria* species identification in domestic rabbits is based on their morphological characteristics, such as oocyst shape, size, the presence or absence of oocyst residue, the presence or absence of a micropyle, and sporulation time. However, this method is inherently subjective [4,5,6,16]. Due to environmental variations, oocysts may undergo morphological changes during development, making accurate species identification challenging [6]. Although oocyst morphology allows for a general differentiation of species, it is practically impossible to analyze thousands of oocysts to confirm the presence of a particular species.

One of the most widely used diagnostic methods for species identification is polymerase chain reaction (PCR), which amplifies millions of copies of a specific DNA fragment in vitro using species-specific oligonucleotide primers [17]. However, due to the sensitivity of these tests, false-positive results caused by contaminants are always a possibility. Recently, various DNA-based molecular differentiation methods using different molecular markers, such as ITS1, ITS1-5.8S, rRNA-ITS2, and 18S rRNA, have been proposed for the identification of *Eimeria* species in rabbits. These methods are more sensitive and less subjective [3,4,6,18,19,20]. PCR amplification of the ITS-1 region has been used to identify different *Eimeria* species in domestic rabbit farms [6,19,20].

The objective of the present study was to identify *Eimeria* species present in domestic rabbits (*Oryctolagus cuniculus*) in Romania, based on morphological characteristics and molecular biology methods.

## 2. Materials and Methods

### 2.1. Morphological Identification

This study included a total of 236 fecal samples collected from rabbits reared in individually caged housing systems within household settings between September 2023 and July 2024. The rabbits were mix-breeds, between two and four months old. Due to the housing conditions (cages with permanent bedding), the prevalence of *Eimeria* infection had been significant in previous years. The rabbits had never undergone deworming treatments. Fecal samples were collected immediately after excretion, placed in properly labeled sterile coproculture containers, stored at 4 °C, and processed within 24–48 h after collection. Standard parasitological screening methods, namely the Willis flotation technique, were used to determine the positivity of fecal samples. The collection, purification, and lysis of oocysts was performed according to standard procedures described by Jackson (1964) and Loug (1976) [21,22].

Following methodologies described in previous studies [4,23,24,25,26,27,28,29], the microscopic morphological identification of sporulated oocysts was carried out by measuring their length (L) and width (W), assessing the presence or absence of a micropyle (M), micropyle cap, oocysts residuum, Stieda body, and determining the sporulation time.

Suspended oocysts were centrifuged at 3100× *g* for 10 min to sediment the oocysts. To facilitate the release of genetic material and ensure efficient DNA extraction from oocysts, the fecal samples were subjected to a physico-chemical pretreatment. The supernatant was completely removed, and the sediment was resuspended in 10 µL of sodium hypochlorite (NaClO, 8%) and incubated at 4 °C for 1.5 h to weaken and partially disrupt the oocyst wall prior to the DNA extraction step. After incubation, the samples were mixed with 35 µL of supersaturated saline solution (NaCl) and incubated at 55 °C for an additional 1.5 h. Following centrifugation at 3100× *g* for 5 min, both the supernatant and sediment were examined under an optical microscope [30].

### 2.2. PCR

Molecular identification was performed through DNA extraction, amplification, and sequencing of the ITS1 gene. DNA extraction was conducted using the MAG MAX Core Nucleic Acid Purification Kit (Applied Biosystems™, Waltham, MA, USA), following the manufacturer’s recommendations. For amplification, the protocol described by Oliveira (2010 [6]) was used, employing 11 pairs of *Eimeria*-specific primers (Table 1). Amplification was carried out using a MyCycler thermal cycler (BioRad^®^, Hercules, CA, USA) under a program consisting of 40 amplification cycles, with an initial DNA denaturation at 95 °C for 1 min, followed by denaturation at 95 °C for 30 s, annealing at 53 °C for 30 s, extension at 72 °C for 30 s, and a final extension at 72 °C for 7 min. PCR results were visualized on a 1.5% agarose gel using the fluorescent dye RedSafe™ (iNtRON Biotechnology, Seongnam-si, Republic of Korea), at a voltage of 120 V and 90 mA for 60 min. To determine the fragment sizes of the amplified products, a 100 bp DNA Ladder marker was loaded into the first well of the gel. After electrophoresis, the gel was analyzed and photographed using a UV transilluminator (UVP^®^, Upland, CA, USA).

For sequencing, the PCR products were purified using the α+ Solution™ GEL/PCR Purification Kit (Alpha-gen, Tantou Rd., Changzhi Township, Pingtung County 908, R.O.C.). The purified PCR products were bidirectionally sequenced (forward and reverse) by Macrogen Europe B.V., Amsterdam, The Netherlands. The sequencing results were compared with the GenBank database using BLAST analysis (BLAST+ 2.16.0) [31].

### 2.3. Statistical Assessment

Statistical analysis was performed using the Fisher’s exact test in GraphPad Prism 9.2.0 (Boston, MA, USA). A difference was considered statistically significant when the *p*-value was less than 0.05 (*p* < 0.05). Confidence intervals for sensitivity and specificity were produced with the Newcombe–Wilson score method [32]. Confidence intervals for positive and negative likelihood ratios were calculated with the method described by Simel and colleagues [33]. The confidence interval for the diagnostic odds ratio was calculated as described by Armitage and Berry [34].

## 3. Results

### 3.1. Morphological Results

**Figure 1:**
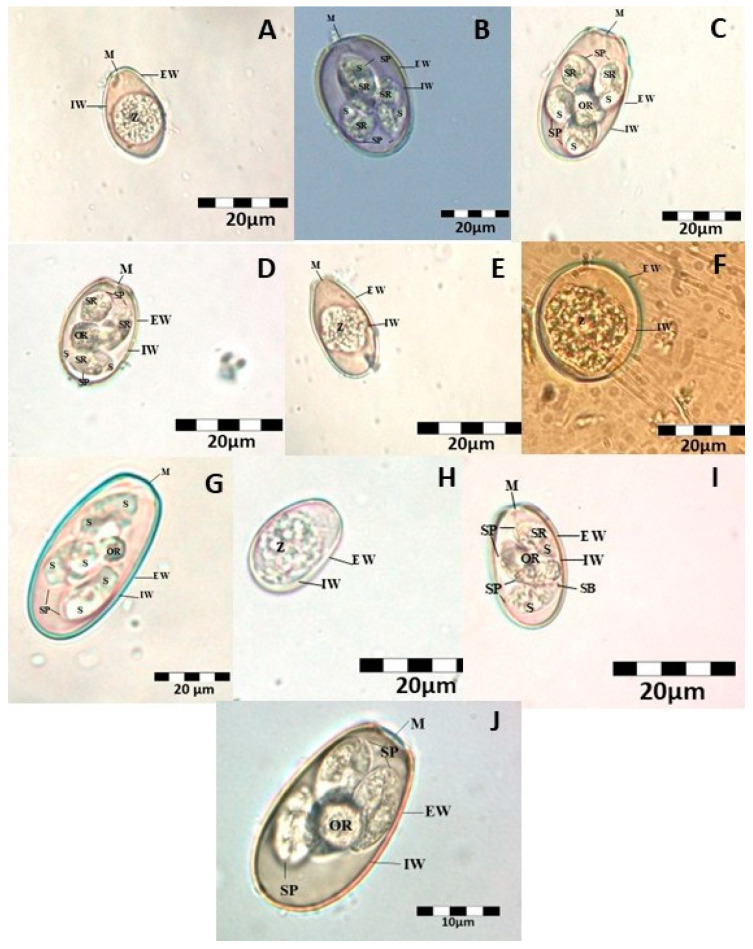
(**A**)-*Eimeria intestinalis*, (**B**)-*Eimeria flavescens*, (**C**)-*Eimeria magna*, (**D**)-*Eimeria media*, (**E**)-*Eimeria piriformis*, (**F**)-*Eimeria exigua*, (**G**)-*Eimeria coecicola*, (**H**)-*Eimeria perforans*, (**I**)-*Eimeria vejdovskyi*, and (**J**)-*Eimeria stiedae* (abbreviations: M-micropyl, EW-external wall, IW-inner wall, Z-zygote, S-sporozoite, SP-sporocyst (s), OR-oocyst residuum, SR-sporocyst residuum, and SB-stieda body).

Out of the 236 fecal samples collected, 183 (77.56%) tested positive for at least one *Eimeria* species. Based on the morphological examination, ten *Eimeria* species were identified: *Eimeria intestinalis* (68.6%), *Eimeria flavescens* (8.9%), *Eimeria magna* (49.2%), *Eimeria media* (7.2%), *Eimeria piriformis* (26.3%), *Eimeria exigua* (49.2%), *Eimeria coecicola* (7.2%), *Eimeria perforans* (7.2%), *Eimeria vejdovskyi* (7.2%), and *Eimeria stiedae* (8.9%) (Figure 1).

Fifty sporulated oocysts were analyzed and measured with the help of a calibrated micrometer. All measurements were performed for each *Eimeria* species in order to ensure precise species identification. Measurements were made in micrometers and presented as averages followed by an interval in brackets (Table 2).

### 3.2. Molecular Screening

For molecular analysis, 20 positive fecal samples were randomly selected from those that showed the highest parasitic load, estimated based on the density of oocysts observed microscopically. This sampling strategy aimed to ensure a sufficient amount of good-quality parasitic DNA, necessary for obtaining relevant results in PCR amplification reactions. The amplification of positive samples was subsequently performed for each of the 11 *Eimeria* species using species-specific primers. The amplification results identified the following species: *E. intestinalis*, *E. flavescens*, *E. vejdovskyi*, and *E. stiedae.* Specifically, nine samples tested positive for *E. intestinalis*, five for *E. flavescens*, four for *E. vejdovskyi*, and two for *E. stiedae*. The sequences obtained in this study have been deposited in GenBank under the following accession numbers: PQ452221–PQ452223 for *Eimeria intestinalis*, PQ452287–PQ452290 for *Eimeria vejdovskyi*, PQ452293–PQ452297 for *Eimeria flavescens*, and PQ453596–PQ453597 for *Eimeria stiedae.* These sequences show a high similarity with the following GenBank entries: *Eimeria intestinalis*—JX406874.1, MK548293.1, MK584296.1, MK584291.1, KX379236.1, OP376063.1, MN645440.1, HM768884.1, MN645341.1, MK584297.1; *Eimeria stiedae*—JQ328190.1, HM768890.1; *Eimeria flavescens*—HM768891.1, OP376060.1, KX379242.1, MT982790.1; and *Eimeria vejdovskyi*—KX379235.1, MN535227.1, HM768883.1, JX406873.1. The comparative analysis of the obtained sequences revealed the presence of multiple distinct haplotypes within each of the four investigated species (*Eimeria intestinalis*, *E. stiedae*, *E. vejdovskyi*, and *E. flavescens*). The differences observed in the nucleotide sequences indicate intraspecific genetic variability, which supports the existence of significant genetic diversity within the studied *Eimeria* populations. This variability may have important implications for understanding the epidemiology, pathogenicity, and resistance to antiparasitic treatments.

### 3.3. Statistical Analysis

Regarding the statistical analysis based on the *p*-value, where a result is considered statistically significant if *p* ≤ 0.05, no statistically significant differences were observed for the samples analyzed in this study (Table 3). A possible reason for the absence of statistically significant differences could be the limited resolution provided by the small set of PCR-analyzed samples, which reduced the statistical power of the analysis and limited the ability to detect subtle but potentially relevant associations between variables.

## 4. Discussion

Coccidiosis remains a serious issue in many countries worldwide, being responsible for high mortality rates among rabbits. This disease is characterized by varying levels of pathogenicity and an affinity for distinct parts of the intestine, making it essential to understand the biology of these parasites.

In Europe, there has been an increased interest in rabbit coccidiosis. In France, a study identified different *Eimeria* species and investigated their pathogenicity levels and localization within the gastrointestinal tract. The most pathogenic species are considered to be *E. intestinalis* and *E. flavescens*, which are intestinally localized, followed by *E. stiedae*, which is hepatic. Although the less pathogenic species do not have a high mortality rate, they significantly impact weight gain and cause severe diarrhea [40]. All the animals in this study were infected with at least one *Eimeria* species. However, the lower number of species identified by PCR compared to microscopic examination suggests that parasitic load and the DNA quantity of each species play a crucial role in the sensitivity of molecular methods. The limited number of species identified from the 20 positive samples can be attributed to several factors. Firstly, the reduced diversity of identified species may reflect the natural dominance of certain more common pathogenic species, such as *E. intestinalis* or *E. magna*, depending on environmental conditions, host age, immune status, and farm type. Secondly, the sensitivity of the detection method used (morphological or molecular) can influence the ability to detect all the present species, especially in cases of mixed infections with low parasitic load for some species. To improve this aspect, the identification method could have been complemented with more sensitive molecular techniques, such as qPCR, which can detect species with low abundance or those in early developmental stages.

The results obtained in this study, which highlight the presence of multiple distinct haplotypes within the same species, are consistent with findings in the scientific literature regarding the genetic diversity of *Eimeria*. This genetic variability has important implications for understanding epidemiology, pathogenicity, and, most notably, for the development of effective control strategies, such as vaccines. In a global context marked by increasing pressure to reduce the use of anticoccidial drugs in animal production, the development of safe and cost-effective vaccines has become essential. However, the success of such vaccines largely depends on a precise understanding of the genetic and antigenic diversity present in field populations of *Eimeria* [41].

Recent studies, particularly those based on single nucleotide polymorphism (SNP) analysis, have revealed considerable diversity among *Eimeria* species, notably in *E. tenella*, one of the most economically important species [42]. These observations underscore the importance of detailed molecular characterization of local isolates in order to better understand population structure and to support the development of effective prophylactic and control measures.

Mixed infections with multiple *Eimeria* species were the most frequently observed in this study, which is consistent with previous research [8,37]. The morphological and morphometric characteristics of both sporulated and unsporulated oocysts of each *Eimeria* species observed in this study are consistent with previously reported findings [8,23,26,27,28,29,35,36,37,38,43,44]. In the present study, *Eimeria exigua* lacked a micropyle, a result similar to those reported by Pellerdy (1974), El-Shahawy (2012, 2018), Abdel-Baki (2013), Duszynski (2013), El-Sayed (2020), and Rabie (2021) [8,23,27,28,36,37,38].

The oocyst residue was present in *E. magna*, *E. media*, *E. coecicola*, *E. vejdovskyi*, and *E. stiedae*, findings that are consistent with those reported by Rabie et al. (2021) [8]. In 2013, Abdel-Baki also observed the presence of oocyst residue in *E. stiedae* [37]. Additionally, sporocyst residue was observed in *E. magna*, *E. media*, *E. flavescens*, and *E. vejdovskyi* in this study. Rabie et al. (2021) similarly noted sporocyst residue in *E. magna* and *E. media* [8]. However, El-Shahawy and El-Goniemy (2018), El-Shahawy et al. (2012), and Kasim and Al-Shawa (1987) did not mention the presence of sporocyst residue in their studies [27,35,37].

To date, 15 *Eimeria* species have been identified in rabbits, with 14 species infecting the intestines and only 1 species (*Eimeria stiedae*) parasitizing the bile ducts. The following *Eimeria* species have been described in the literature: *E. perforans*, *E. piriformis*, *E. exigua*, *E. media*, *E. magna*, *E. coecicola*, *E. vejdovskyi*, *E. flavescens*, *E. intestinalis*, *E. nagpurensis*, *E. irresidua*, *E. matsubayashi*, *E. elongata*, and *E. neoleporis*. However, only eight of these species are considered economically significant [45]. *Eimeria* species infecting domestic rabbits have been reported in multiple countries, including France, the United Kingdom, Egypt, Brazil, Iran, Indonesia, and China [4,26,27,46,47,48,49].

In the present study, the overall prevalence of *Eimeria* infestation in domestic rabbits from the four counties was 77.56% (183/236). This highlights the importance of monitoring and controlling *Eimeria* spp. infections in rabbit farms to prevent their negative impact on health and productivity. The infection rate observed in this study was higher than those reported in Brazil (19.5%, 100/514) [48], Iran (31.0%, 22/71) [26], Egypt (70.0%, 70/100) [27], and Indonesia (70.3%, 527/750) [49], but lower than that reported in the United Kingdom (96.0%, 572/596) [46]. Furthermore, compared to studies conducted in different regions of China, the prevalence observed in this study was lower than that reported in Xinjiang (100.0%, 785/785) [24] and Weifang (83.9%, 141/168) [50], but higher than those reported in Shandong (20.0%, 123/616) [51], Fujian (44.0%, 400/909) [52], Henan (44.2%, 152/344) [53], and Sichuan (56.4%, 62/110) [54]. In a study conducted in Germany, *Eimeria media*, *E. magna*, and *E. perforans* were the most frequently detected species, while the fourth identified species, *E. exigua*, was relatively rare [55]. The first three species mentioned are also among the most common *Eimeria* species found in fattening rabbit farms, as previously reported in various countries such as France, Belgium, Italy, and England [46,56,57,58].

In the molecular analysis, the ITS1 marker specific to *Eimeria* spp. was used for PCR amplification. Previous studies have employed different molecular markers, such as ITS1-5.8S, rRNA-ITS2, and 18S rRNA. Kvicerova [4] obtained molecular data on 18S rRNA and COI fragments by injecting approximately 20 individually collected oocysts from rabbits. Oliveira [6] obtained molecular data on ITS-1 fragments by reproducing 11 *Eimeria* isolates in rabbits. Yan [3] obtained molecular results on ITS1-5.8S rRNA-ITS2 fragments by inoculating oocysts into 40-day-old rabbits. Heker [46] amplified genomic DNA from domestic rabbit fecal samples using PCR to obtain molecular data on ITS-1 fragments. 18S rRNA sequences, combined with ITS regions, have been considered useful genetic targets for species-level identification [6,59,60,61]. In the present study, the ITS1 sequence similarity ranged between 94% and 99% for *Eimeria intestinalis*, 97% and 100% for *Eimeria stiedae*, 91% and 98% for *Eimeria flavescens*, and 94% and 99% for *Eimeria vejdovskyi*. The 18S rRNA sequence similarity among the 11 rabbit *Eimeria* species varied from 92.7% (between *Eimeria* n. sp. and *E. stiedae*) to 98.8% (between *Eimeria* n. sp., *E. piriformis*, and *E. perforans*) [62]. In 2012, five rabbit *Eimeria* species were isolated in China, and their ITS1 sequences were compared with rabbit *Eimeria* species stored in GenBank, revealing a within-species sequence similarity of over 96.9% [63].

## 5. Conclusions

The present study highlights the necessity of combining morphological and molecular identification methods for a more accurate identification of *Eimeria* spp., which can contribute to more precise diagnostics and the development of more effective strategies for controlling coccidiosis in rabbits. Coccidiosis caused by *Eimeria* spp. is a common parasitic disease in rabbits, with a high infection rate, as demonstrated by the 77.56% positivity rate among the analyzed samples. Molecular biology techniques, utilizing the ITS-1 rRNA marker, confirmed the presence of four *Eimeria* species. However, not all species present in a sample could be identified through PCR, most likely due to variable parasitic loads and insufficient DNA quantities per species. Although microscopic identification of oocysts enabled the detection of ten *Eimeria* species, this method has limitations in terms of identification accuracy.

## Figures and Tables

**Table 1:** Primer sequences used for genetic amplification of ITS1 *Eimeria* spp. and for each of the 11 *Eimeria* species affecting hares (courtesy of Oliveira, 2010 [6]).

Species	Primer Name	Sequence	Amplicon Size (bp)
*Eimeria* spp. (all species)	ITS1-F ITS1-R	GGGAAGTTGCGTAAATAGA CTGCGTCCTTCATCGAT	400–600
*E. magna*	Emag-ITS1-F Emag-ITS1-R	TTTACTTATCACCGAGGGTTGATC CGAGAAAGGTAAAGCTTACCACC	218
*E. coecicola*	Ecoe-ITS1-F Ecoe-ITS1-R	AGCTTGGTGGGTTCTTATTATTGTAC CTAGTTGCTTCAACAAATCCATATCA	256
*E. exigua*	Eexi-ITS1-F Eexi-ITS1-R	GAATAAGTTCTGCCTAAAGAGAGCC TATATAGACCATCCCCAACCCAC	280
*E. flavescens*	Efla-ITS1-F Efla-ITS1-R	GAATATTGTTGCAGTTTACCACCAA CCTCAACAACCGTTCTTCATAATC	199
*E. intestinalis*	Eint-ITS1-F Eint-ITS1-R	TGTTTGTACCACCGAGGGAATA AACATTAAGCTACCCTCCTCATCC	241
*E. irresidua*	Eirr-ITS1-F Eirr-ITS1-R	TTTGGTGGGAAAAGATGATTCTAC TTTGCATTATTTTTAACCCATTCA	226
*E. media*	Emed-ITS1-F Emed-ITS1-R	GATTTTTTTCCACTGCGTCC TTCATAACAGAAAAGGTAAAAAAAGC	152
*E. perforans*	Eper-ITS1-F Eper-ITS1-R	TTTTATTTCATTCCCATTTGCATCC CTTTTCATAACAGAAAAGGTCAAGCTTC	157
*E. piriformis*	Epir-ITS1-F Epir-ITS1-R	ACGAATACATCCCTCTGCCTTAC ATTGTCTCCCCCTGCACAAC	289
*E. stiedae*	Esti-ITS1-F Esti-ITS1-R	GTGGGTTTTCTGTGCCCTC AAGGCTGCTGCTTTGCTTC	217
*E. vejdovskyi*	Evej-ITS1-F Evej-ITS1-R	GTGCTGCCACAAAAGTCACC GCTACAATTCATTCCGCCC	166

**Table 2:** Comparative Morphological and Morphometric Characteristics of *Eimeria* Oocysts from Domestic Rabbits (*Oryctolagus cuniculus*) in the Present Study with Previously Described Data.

Species	Oocyst		Micropyle	Micropyle Cap	Shape	Oocysts Wall Color	Oocysts Residuum	Stieda Body	Sporulation Time	References
	Length (µm)	Width (µm)								
*Eimeria* *coecicola*	27.44 (25.89–30.07) 38.8 (33–44) 33.4 (28–40) 24 (23–29) 35.5 (23–40) 30.52 28.10 29.75 (27.41–31.58)	13.37 (12.02–15.88) 19.8 (16–23 19.3 (15.5–22) 14 (11–17) 19.5 (15–21) 17.56 15.64 17.38 (16.27–18.11)	+ + + + + + + +	− − − −	Elliptic Cylindrical Cylindrical Cylindrical Elliptic Elliptic Elliptic Cylindrical	Greenish Yellowish Yellowish green Yellowish green Light yellow Yellowish green	+ + + + + + + +	− −	60 h 56 h 56 h 62 h 62 h 60 h 60 h	Present study [23] [35] [36] [28] [37] [38] [8]
*Eimeria* *exigua*	12 (10.21–16.76) 14.5 (10–18) 15 (14–17) 15 (14–17) 14 (12–21) 15.83 14.64 19.41 (17.13–24.27)	12 (10.21–16.76) 12.7 (9–16) 15 (14–17) 15 (14–17) 13 (9–18) 15.83 13.57 19.61 (17.56–23.37)	− − − − − − − −	− − − −	Sphere Sphere Sphere Sphere Sphere Sphere Sphere Sphere	Yellowish green Purple Purple Yellow Purple	− − − − − − − −	− −	45 h 20 h 56 h 36 h 28 h 20 h 45 h	Present study [23] [27] [36] [28] [37] [38] [8]
*Eimeria* *flavescens*	26.54 (23.46–35.52) 31.7 (25–37) 32 (27–37.5) 23 (22–30) 23 (22–30) 31.7 (25–37) 29.22 27.83 29.63 (24.71–34.42)	19.07 (17.05–24.37) 21.4 (14–24) 21.2 (17–25) 16.2 (14–18) 16 (14–18) 21.4 (14–24) 16.7 19.44 21.08 (17.06–25.45)	+ + + − + + + + +	− − −	Oval Oval Oval Oval Oval Oval Oval Oval Oval	Greenish brown Yellow Light brown Brown Brown Yellow Yellowish brown	− − − − − − − − −	−	45 h 38 h 38 h 50 h 40 h 38 h 56 h 51 h 45 h	Present study [39] [35] [27] [36] [28] [37] [38] [8]
*Eimeria* *intestinalis*	28.61 (25.64–29.33) 27 (27–32) 29.2 (25–34) 20.3 (18–23) 20 (19–24) 27 (21–36) 28.29 25.71 25.19 (23.38–26.56)	18.44 (16.21–19.97) 18 (17–20) 19.3 (17–22) 13.5 (12–15) 13 (12–15) 18 (15–21) 17.08 18.60 18.3 (16.81–19.37)	+ + + + + + + + +	− − − −	Piriform Piriform Piriform Piriform Piriform Piriform Piriform Piriform Piriform	Yellowish Yellowish Greenish brown Greenish brown Greenish brown Yellow brown Greenish brown	− + + + + + + + +	− −	52 h 48 h 60 h 50 h 48 h 55 h 58 h 52 h	Present study [23] [35] [27] [36] [28] [37] [38] [8]
*Eimeria* *magna*	37.75 (35.64–40.31) 35 (31–40) 35.4 (29–40) 24 (23–26) 22 (19–24) 35 (27–41) 28.64 28.41 27.44 (25.34–29.4)	22.51 (20.01–25.36) 24 (22–26) 24.2 (21–26.5) 14.3 (13–16) 12 (10–15) 24 (17–29) 16.7 19.23 18.61 (16.36–22.11)	+ + + + + + + + +	+ + − −	Oval Oval–Elliptic Oval Oval Elliptic Oval–Elliptic Oval-Elliptic Oval Oval	Yellowish brown Dark yellow Yellowish brown Red brown Brownish red Dark yellow Yellowish brown	+ + + + + + + + −	− +	30 h 48 h 45 h 30 h 44 h 36 h 43 h 31 h	Present study [23] [35] [27] [36] [28] [37] [38] [8]
*Eimeria* *media*	31.86 (29.26–35.37) 31.2 (27–36) 30 (25.5–34) 22.3 (19–24) 22 (19–24) 31.2 (27–36) 28.64 27.48 27.44 (25.34–29.4)	22.82 (18.17–24.69) 18.5 (15–22) 18.7 (15–22) 12.1 (10–15) 12 (10–15) 18.5 (15–22) 16.7 17.79 18.61 (16.36–22.11)	+ + + + + + + + +	− − + −	Oval–Elliptic Oval Oval–Elliptic Oval–Elliptic Elliptic Oval–Elliptic Oval–Elliptic Oval–Elliptic Elliptic	Light pink Light pink Light pink Yellow Yellow Delicate pink Light pink	+ + + + + + + + +	− −	30 h 36 h 36 h 30 h 36 h 36 h 34 h 31 h	Present study [23] [35] [27] [36] [28] [37] [38] [8]
*Eimeria* *perforans*	19.18 (16.45–25.85) 22.7 (15–29) 20.9 (14–27) 15.6 (12–18) 16 (13–18) 23 (15–31) 22.16 18.36 24.19 (16.46–26.68)	12.96 (10.75–15.22) 14.2 (11–17) 13.7 (11–19.5) 10.3 (8–11) 10 (9–11) 14 (11–20) 14.45 12.65 17.08 (11.73–19.76)	− + − + + − − −	− − − −	Elliptic Elliptic Elliptic Elliptic Elliptic Oval Oval Oval–Elliptic Elliptic	Light brown Clear Greenish Greenish Greenish Delicate pink Greenish	− + + + + + + + +	− +	30 h 24 h 25 h 25 h 36 h 36 h 22 h 30 h	Present study [23] [35] [27] [36] [28] [37] [38] [8]
*Eimeria* *piriformis*	33.45 (31.58–35.79) 29 (26–32) 26 (24–32) 29 (26–33) 27.87 23.98 (22.2–25.12)	17.87 (15.47–19.86) 18 (17–21) 20 (19–21) 18 (17–21) 17.97 18.25 (17.93–18.51)	+ + + + + +	− − + −	Piriform Piriform Piriform Piriform Elliptic Piriform	Yellowish Yellowish brown Tan Yellowish brown yellowish	− − − − − −	− −	40 h 28 h 27 h 36 h 40 h	Present study [23] [36] [28] [37] [8]
*Eimeria* *stiedae*	39.69 (37.41–40.98) 37 (28–40) 26.5 (24–29) 26 (25–29) 37 (31–42) 28.13 (27.65–28.44)	22.45 (19.74–24.63) 21 (16–25) 13.1 (11–15) 13 (12–15) 20 (17–25) 16.99 (16.55–17.66)	+ + + + + +	+ − +	Oval Oval Elliptic Elliptic Oval Oval	Salmon Salmon Light brown Pink Pink	+ − + + + −	− +	58 h 55 h 60 h 60 h 58 h	Present study [23] [27] [36] [28] [8]
*Eimeria* *vejdovskyi*	29.55 (25.46–32.85) 29.05 (24–33) 32.9 (30–37) 28.64 (27.27–30.44)	17.14 (15.22–20.37) 18.18 (15–20) 19.2 (18–21) 18.97 (18.08–20.27)	+ + + +	− −	Elliptic Cylindrical Elliptic Oval	Yellowish Yellowish	+ + +	−	48 h 48 h 48 h 48 h	Present study [5] [28] [8]

**Table 3:** Fisher’s test statistical analysis based on the distribution of *Eimeria* species infestations in rabbits.

Sample No.	Species	N * (% **)	OR ^★^ (95%CI ^★★^)	*p* Value
Positive	*-*	183 (77.54%)	0.7754 (0.7180–0.8240)	-
Positive for one species	*E. intestinalis*	29 (15.85%)	0.1585 (0.1127–0.2183)	0.115
Positive for two species	*E. stiedae*, *E. flavescens*	21 (11.48%)	0.1148 (0.0763–0.1691)	0.159
Positive for three species	*E. intestinalis*, *E. magna*, *E. exigua*	54 (29.51%)	0.2951 (0.2338–0.3648)	0.295
Positive for four species	*E. magna*, *E. exigua*, *E. piriformis*, *E. intestinalis*	62 (33.88%)	0.3388 (0.2742–0.4101)	0.339
Positive for five species	*E. media*, *E. coecicola*, *E. perforans*, *E. intestinalis*, *E. vejdovskyi*	17 (9.29%)	0.0929 (0.0588–0.1437)	0.093

* N—no. of positive samples, ** Prevalence, ^★^ OR—odds ratio, and ^★★^ CI—confidence interval, *p* < 0.05.

## Data Availability

Data are contained within the article.
